# Medical Topography of Brazil and Uruguay; with Incidental Remarks

**Published:** 1845-02

**Authors:** 


*Medical Topography of Brazil and Uruguay; with Incidental Remarks.* By G. R. B. Horner, M. D., Surgeon U. S. Navy, Honorary Member of the Philadelphia Medical Society, and Corresponding member of the National Institute at Washington. 8vo. pp. 296. Philadelphia, Lindsay & Blakiston: 1845.

The information contained in this work is derived from the author's personal observations during two cruises in the U. S. Ships Macedonian and Delaware, in the years 1841 and 1842.

The region of country visited by him possesses many objects of interest for the naturalist, the physician, and the general inquirer. The boldness of its scenery; the richness and variety of its productions; its commercial relations; institutions, civil and religious; customs of the inhabitants, &c.—with all of which the people of this country are but little informed—are subjects of interesting inquiry. In the hasty glance we have given to the book, we have been more particularly interested in the account of the productions of the soil, and the medical institutions of Brazil. In reference to the latter, it is our purpose to give some extracts from the work in a future number, that our readers may know something of the paternal care evinced by the government of that remote country, in a matter which so nearly concerns the humblest as well as the highest members of every community.

We observe that Dr. Horner has dedicated his book to Dr. John Bell, of this city—a just tribute to learning and abilities.

In mechanical execution, this is a beautiful volume—the binding, paper, typography, illustrations—all are creditable to the Philadelphia press.

